# The Duty of the Physician to the School Child

**Published:** 1908-01

**Authors:** 


### THE DUTY OF THE PHYSICIAN TO THE SCHOOL CHILD.

From his survey of the condition of the child in our public schools, Hollopeter draws the following conclusions:

The school life of a child at the present day is too complex and difficult. Too many subjects for study have been introduced, and too great a thoroughness required for a young mind. This has a tendency to unbalance development and create nervous irritability.

In teaching large classes the personal equation is lost. The most valuable element of the teacher is showered on the bright child, while the backward or defective are frequently lost to sight.

Teachers who fail to recognize a defective child commit a great injury by permitting the child's mind to be unemployed. This is especially true of the depraved type of children. The children in our reform schools, the great army of the police court, chronic drunkards and criminals, the tramp, vagrants, low prostitutes, are largely recruited from this class of the slightly mentally deficient who were neglected in their youth.

In the gathering of classes of children of unequal capacity, the teacher fails to recognize the varied powers of attention, which is of first importance in the process of mental development.

The physician should take a deeper interest in watching the mental defects and having children so afflicted properly classified. The physical defects, including those of eye, ear, nose, should receive more attention than is now given.

We as a profession are confronted with the appalling information that 120,000 defective school children exist in the United States.—*Jour. A. M. A., Oct. 19th. '07.*

